# Urinary Incontinence: An Update

**DOI:** 10.1155/2019/5707659

**Published:** 2019-07-01

**Authors:** Dragana Zivkovic, Vladimir Kojovic, Damir Franic

**Affiliations:** ^1^Institute for Child and Youth Health Care of Vojvodina, Novi Sad, Serbia; ^2^Faculty of Medicine, University of Novi Sad, Serbia; ^3^Institute for Mother and Child “dr Vojkan Cupic” Belgrade, Serbia; ^4^Faculty of Medicine, University of Belgrade, Serbia; ^5^Outpatient Clinic Ob & Gyn Rogaška Slatina, Slovenia; ^6^Faculty of Medicine, University of Maribor, Slovenia

Urinary incontinence is a medical condition characterized by involuntary loss of urine that occurs during the day, or during the night. It is a global health issue which affects 400 million citizens worldwide. There are several forms of urinary incontinence: stress incontinence, urge incontinence, mixed incontinence, and nocturia. Regardless of the form and severity of the condition, it affects patient's daily life, and it can have repercussions on their physical, financial, social, and emotional well-being. Not an irrelevant issue is a negative influence on their sexual health.

It is not a simple condition with straightforward treatment protocols. This condition is also treated by a number of different medical and nonmedical professionals, alone, or as members of a multidisciplinary team. Deciding on a treatment regimen is a multifactorial decision process dictated in part by urinary incontinence subtype and symptom severity.

Our aim as the editors was to cover the problem of urinary incontinence from the gynecological, urological, pediatric, and physiotherapeutic perspective. After careful evaluation of the submitted articles, 9 were selected for publication. All of the articles were either gynecologic (6) or urologic (3) topics.

In a paper by T. Rechberger et al., the authors found that treatment with solifenacin or mirabegron may significantly reduce the incidence of undesired lower urinary tract infections after midurethral sling operation. Therefore, the prevalence of urgency and frequency episodes one week after sling placement were significantly reduced.

In the prospective study by E. Malanowska et al., the authors wanted to figure out if surgical repair with laparoscopic uterine lateral suspension could improve the symptoms of overactive bladder (OAB). In their study the OAB resolution after the procedure was found in 76% of women, while de novo OAB was present in 2.6%.

On the other hand, S. Ciećwież et al. showed in their prospective observational study that “de novo” onset of OAB is more frequent in patients after Burch suspension than in patients after the sling operations, due to the postoperative bladder volume reduction after Burch suspension.

Similar topic was covered by J. H. No et al. They studied the modalities of prediction of OAB in patients following midurethral sling operations (REEMAX). They found out that reduced peak urinary flow rate after the procedure could be a promising and noninvasive metric procedure which could predict the onset of OAB.

The randomized trial performed by M. Ptak et al. showed that the combined training of the pelvic floor muscles (PFM) and the synergistic transversus abdominis muscle improves the quality of life of women with stress urinary incontinence (SUI). The exercises for the PFM and the synergistic muscles give better results in women who have given birth fewer than three times than isolated PFM exercises.

The review article by D. Franic and I. Fistonic made an overview of up-to-date data of laser treatment possibility in women with SUI and genitourinary syndrome of menopause (GSM). They chronologically stressed the evidence based medicine data concerning laser treatment in women with SUI and GSM and precisely showed which women could be the candidate for laser treatment and how important is to select the women where laser treatment could be a therapy of choice for treating SUI or GSM. Moreover, very important was to introduce the laser as a potential therapy of choice for treating GSM in breast cancer survivors. Nevertheless, they also conclude how important is to follow up the patients after the procedure and emphasized the importance of long-term well-designed prospective studies to disclose the effectiveness of laser in SUI and GSM treatment.

P. Miotla et al. evaluated the effectiveness of a phytotherapic drug (Canephron N) in preventing urinary tract infection in high-risk women undergoing urodynamic studies (UDS). They have showed that prophylaxis of UTI with a phytodrug (Canephron N) may be considered as a good alternative to antibiotic prophylaxis use after UDS in high-risk female patients.

A paper by S. H. Kim et al. describes an alternative option for measuring the total prostate volume (TPV) in patients with colorectal cancer where standard transrectal ultrasonography (TRUS) is not an option. The authors estimated the correlations of the TPV measurements made using computed tomography (CT), magnetic resonance imaging (MRI), and TRUS in 122 patients. They found that, when stratified by a prostate size of 30 mL, TRUS and CT or MRI did not correlate well with the prostate size < 30 mL; however, CT and MRI had a better correlating power for prostate size ≥30 mL. The authors concluded that preoperative MRI is the best alternative modality for TPV measurement for the patients who cannot undergo a TRUS assessment.

T. Y. Shin and Y. S. Lee presented a prospective study of 95 patients who underwent robot-assisted radical prostatectomy (RARP) and analysed whether their postoperative continence rates depend on surgical technique of detrusor repair. Standard RARP was performed in forty five patients, and their detrusor was closed by standard approximation of resected detrusor muscle. RARP using novel detrusorrhaphy technique was performed in fifty patients. The point of the detrusorrhaphy technique is “zig–zag” suturing which aims to reconstruct, thicken, and strengthen intraoperatively damaged detrusor in a physiologically and anatomically ideal way. The authors concluded that detrusorrhaphy technique is simple, safe, and feasible, with significantly better outcomes than those achieved with the standard RARP technique in terms of urinary incontinence.

J. van Uhm et al. presented minimally invasive treatment of postprostatectomy incontinence using Opsis, injectable bulking agent. The authors analyzed 10 patients with minimal urine loss (<30gr/24h). Nine patients had unsuccessful treatment, and only one patient improved his incontinence. Moreover, four patients reported complications as urinary frequency and hematuria. This pilot study revealed that treatment of urinary incontinence with Opsis bulking agents is not an effective option and it can lead to the worsening of incontinence symptoms.

We believe that the articles in this special issue give a valuable perspective on different aspects of urinary incontinence.

